# How we remember what we can do

**DOI:** 10.3402/snp.v5.24807

**Published:** 2015-10-26

**Authors:** Gunnar Declerck

**Affiliations:** Sorbonne universités, Université de technologie de Compiègne, EA 2223 Costech (Connaissance, Organisation et Systèmes Techniques), Centre Pierre Guillaumat - CS 60 319 - 60 203 Compiègne cedex, France

**Keywords:** cognitive neuropsychology, phenomenology, action memory, motor simulation, mental imagery, affordance, action possibility

## Abstract

According to the motor simulation theory, the knowledge we possess of what we can do is based on simulation mechanisms triggered by an off-line activation of the brain areas involved in motor control. Action capabilities memory does not work by storing some content, but consists in the capacity, rooted in sensory-motor systems, to reenact off-line action sequences exhibiting the range of our powers. In this paper, I present several arguments from cognitive neuropsychology, but also first-person analysis of experience, against this hypothesis. The claim that perceptual access to affordances is mediated by motor simulation processes rests on a misunderstanding of what affordances are, and comes up against a computational reality principle. Motor simulation cannot provide access to affordances because (i) the affordances we are aware of at each moment are too many for their realization to be simulated by the brain and (ii) affordances are not equivalent to currently or personally feasible actions. The explanatory significance of the simulation theory must then be revised downwards compared to what is claimed by most of its advocates. One additional challenge is to determine the prerequisite, in terms of cognitive processing, for the motor simulation mechanisms to work. To overcome the limitations of the simulation theory, I propose a new approach: the direct content specification hypothesis. This hypothesis states that, at least for the most basic actions of our behavioral repertoire, the action possibilities we are aware of through perception are directly specified by perceptual variables characterizing the content of our experience. The cognitive system responsible for the perception of action possibilities is consequently far more direct, in terms of cognitive processing, than what is stated by the simulation theory. To support this hypothesis I review evidence from current neuropsychological research, in particular data suggesting a phenomenon of ‘fossilization’ of affordances. Fossilization can be defined as a gap between the capacities that are treated as available by the cognitive system and the capacities this system really has at its disposal. These considerations do not mean that motor simulation cannot contribute to explain how we gain perceptual knowledge of what we can do based on the memory of our past performances. However, when precisely motor simulation plays a role and what it is for exactly currently remain largely unknown.

Action memory is not limited to storing and retrieving data about what we did yesterday or during last Christmas, i.e. to episodic memory. It is also in charge of keeping track of our action capabilities. Maintaining a precise knowledge of what it is able to do is essential for any biological system capable of movement. Human beings are no exception. To react correctly to situations, we must be able to anticipate what actions are achievable in our immediate environment. We must consequently possess a precise knowledge of our behavioral capacities and attunements of those capacities to situations, objects, and states of affairs. The memory system keeping track of what we can do is especially involved in the calibration of perceptual contents on our behavioral repertoire – which occurs without the need of engaging into any reasoning or deliberative process – i.e. in the perception of what ecological psychologists call the affordances of objects. To be able to perceive what objects afford, in one way or another we must possess some knowledge of what our body and skills make us capable of doing and what conditions must be fulfilled for this to be done. Obviously, this kind of knowledge is based on the memorization of actions performed in the past and demonstrating the range of our powers, e.g. the operational distance to which one can reach with one's arm, the speed with which one can move to a target or avoid an obstacle, the object width one can cover with the hand span, the weight one can lift, the height of stairs or ground slope one can climb, and so on. That is: we know what we are able to do right now and perceive the environment accordingly because we remember what we were able to do in past circumstances.

In recent years, the motor simulation theory (ST) has proposed a neurocomputational model of how this kind of knowledge is maintained in humans and higher animals such as monkeys. The main claim of ST is that the knowledge we possess of what we can do is based on simulation mechanisms triggered by an off-line activation of brain areas involved in motor control. Motor simulation is what explains the calibration of perceptual content on our behavioral repertoire: we perceive what objects afford because simulation mechanisms enabling the virtual realization of the afforded action are involved in perceptual data processing. These mechanisms would, for instance, enable to calibrate visual distances on the reaching or moving-close-to capabilities of the perceiving agent, thus explaining the functional delimitation between peripersonal and extrapersonal space.

The ST account of action capabilities memorization is consistent with embodied and enactive approaches to cognition, especially when they assume a form of non- (or at least weak) representationalism. These approaches, when applied to memory, are characterized by at least two requirements: (i) rooting memory performances in sensory-motor or, more generally, bodily skills; and (ii) considering the biological nature of memory, i.e. treating memory as a competence of living systems, and taking into account in a realistic way its biological substrate. Those requirements are at least partially filled by the ST framework. According to ST, action capabilities memory does not work by storing some content, but consists in the capacity, rooted in sensory-motor systems, to reenact off-line action sequences. Our knowledge of what we can do is not propositional or symbolic, it is dispositional: we know what we can do insofar as we are able to reenact (virtual) motor episodes exhibiting our capacities and taking into account parameters characterizing real action.

In this paper, I will present several arguments which demonstrate that the neurocomputational mechanisms postulated by ST are not sufficient to explain how we memorize what we can do and use this memory to build perceptual representations. These arguments rely on evidence from cognitive psychology and neuroscience, but also first-person approaches to perception, i.e. phenomenological data. One central motivation behind this critique is related to the conception assumed by ST of what it means to represent (or ‘be aware of’ or ‘know’) something as possible, and what must be done by a cognitive system, in terms of information processing, to be in a position to anticipate the possibilities realizable with an object. What seems to be systematically, yet implicitly, assumed by ST advocates is that any action, to be represented as possible, *must be realized in the mind*. Only running virtually the action can tell you if this action can or cannot be done. This assumption looks sensible at first sight: affordances being merely *possible* actions (they are not yet realized when they are anticipated), how could they have a cognitive reality – be conceived or perceived – if not realized in advance ‘in’ the brain? Whether sensible or not, I am convinced that this assumption is ill-founded. If simulating actions was a prerequisite to anticipate what is possible to do, the possibilities we would be aware of at each moment would be reduced to the few actions we are about to perform (because they correspond to the next step of the course of action into which we are currently engaged), or to the actions we consider perhaps relevant when planning a strategy. This is obviously not the case. At each moment, we are aware of a huge field of possibilities which do not relate to what we are currently doing. This is a key issue that is not accounted for by ST. Most of the action possibilities we are aware of through perception are intrinsic dispositional properties of objects and *are not bound to immediate or even mediate realizability*. The mechanisms described by ST can be used to evaluate the feasibility of actions given the current state, situation, and pragmatic resources of the individual, i.e. to anticipate whether a specified behavior taking advantage of a specified affordance will be a success or a failure given input parameters specifying current circumstances; but what objects afford does not depend on what can be done right now or in the immediate or far future.

To overcome the limitations of ST, I will propose and sketch the basic principles of a new approach: what I call the *direct content specification hypothesis*. This hypothesis, which can be viewed as a phenomenological extension and complement to J.J. Gibson's direct theory of perception (Gibson, 1977, 1979), consists in claiming that, at least for the most basic actions of our behavioral repertoire, the action possibilities we are aware of through perception are directly specified by perceptual variables characterizing the content of our experience. One obvious illustration of such direct content specification is visual perception of distance: visual distance conveys knowledge about what we can do (e.g. whether we can or cannot reach something) but can be specified on the basis of optical cues alone. This is made possible because our past performances (typically the distance our arm can reach) are directly registered using the same perceptual variables which are used in distance perception. The same kind of mechanism is used to calibrate the size of objects using the metric of our hand grip span.

The cognitive system responsible for the perception of action possibilities is consequently far more direct, in terms of cognitive processing, than what is stated by ST. Motor simulation mechanisms certainly contribute to our knowledge of what we can do (or at least to our knowledge of *how* we can do what we can do), but this knowledge also involves more direct perceptual mechanisms, and both are complementary. Motor simulation is probably useful to evaluate if certain categories of planned actions can be realized in current circumstances, but it cannot account for how we gain perceptual knowledge of what we can do based on the memory we keep of our past performances, regardless of whether we plan to do it or not.

## The ST: origins and main theoretical claims

ST constitutes the main explanation currently offered in neuropsychology to account for the ability to anticipate what actions are realizable in a given situation at time *t*, and thus perceive what surrounding objects and structures enable us to do.^1^ The principles of ST were initially developed for modeling the proactive nature of the motor control system. The function of proactive models is to explain how the motor plan can be adjusted before the peripheral signals resulting from movement execution reach the brain (Grush, 2003), i.e. how a feed-forward mode of motor control substitutes to a purely reactive one. The main claim of ST is that movement simulation mechanisms exploiting internal models of the musculoskeletal system are behind this proactive regulation. The simulation-based prediction of the consequences of the motor commands makes possible their anticipated adjustment (see e.g. Blakemore, Goodbody, & Wolpert, 1998; Desmurget & Grafton, 2000; Wolpert, Ghahramani, & Jordan, 1995; Wolpert & Kawato, 1998). This hypothesis takes up the principles of the efference copy model, which postulates the existence of sensorimotor emulation mechanisms anticipating the consequences of efferent signals, but extends its scope; simulation is not only involved during movement execution to enable early corrections of the motor plan, but also before it starts, during the phase of motor planning and decision (Grush, 2003).

Subsequently, various authors proposed to extend this model to the construction of perceptual representations, arguing that similar mechanisms are responsible for (i) the construction of pragmatic or motor representations, which enable us to represent objects as goals for action (Jeannerod, Arbib, Rizzolatti, & Sakata, 1995; Jeannerod & Jacob, 2005), and, more generally, (ii) the perceptual categorization of objects with regard to skills and action capacities of the subject, in other words, the perception of affordances. According to this hypothesis, the perception of the actions potentiated by the environment shares common neurocognitive mechanisms with movement execution and control, resulting from an ‘off-line’ activation of the motor system and associated sensorimotor processing modules (Grush, 2003; Jeannerod, 2001). These mechanisms explain the proactive nature of the behavior; because they enable an anticipated realization of the future, they make it possible to tune one's actions to something that has not yet occurred.

This extended version of ST is sketched by most authors promoting the motor version presented above, as well as in De'Sperati and Stucchi (1997, 2000), but was chiefly systematized by Jeannerod (1994, 2001, 2006), Jeannerod et al. (1995), Gallese (2000), Hesslow (2002), Grush (2003, 2004, 2007), Garbarini and Adenzato (2004), Coello and Delevoye-Turrell (2007), Cisek (2007), Witt and Proffitt (2008), Pezzulo (2008), Moulton and Kosslyn (2009), Pezzulo, Barca, Bocconi, and Borghi (2010), Sinigaglia and Rizzolatti (2011). It is also advocated in the field of robotics by authors such as Möller (1999), Ziemke, Jirenhed, and Hesslow (2005), Hesslow and Jirenhed (2007a, b), Hoffmann (2007), Erdemir et al. (2008), Schenck (2009), Schenck, Hasenbein, and Möller (2012), Dindo et al. (2013). Robotics research attaches particular importance to the role motor simulation can play in determining, given some input conditions, what action or sequence of actions from a repertoire of actions {A_1_,…, A_n_} enable the achievement of a specified desired state (Schenck et al., 2012).

It must immediately be noted that, except in the work of Grush (see e.g. Grush, 2003), what ST puts exactly behind the term ‘simulation’ is generally poorly specified. To clarify the forthcoming discussion, let's try a minimal characterization. In very general terms, a simulation can be defined as a computational mechanism which enables us to model the execution of a process, i.e. a temporal sequence of state changes (see Craik, 1943, chap. 5). Based on input data specifying at time *t*_0_ the value of a series of parameters relevant for characterizing the realization of the to-be-simulated process (the initial state), the simulator calculates the value of these parameters at a given time step *t*_n_ of the execution of the process (the end state or desired state). When applied to motor cognition, the simulated process is a body action, such as the action of reaching and grasping an object, for which relevant parameters are, e.g., the position of the hand relative to the object in an egocentric reference frame or the distance between the fingers used for the grasping action (generally, the thumb and the index finger) relative to the dimensions of the object (Jeannerod, 2003). As Demougeot and Papaxanthis (2011) explain, ‘forward models mimic the causal flow of the physical process by predicting the consequences (e.g. position, velocity) of a motor command’.

Additional features of motor simulation can be highlighted based on its differences with *mental imagery* and *sensorimotor emulation*, with which, unfortunately, motor simulation is frequently confused, especially in the robotic literature. Unlike mental imagery, which is explicit and does not necessarily involve a representation of body activity, motor simulation (a) is most of the time purely implicit and (b) always works with (realistic) models of the body. Unlike sensorimotor emulation, which refers to a blind calculation process of sensory inputs, motor simulation (c) works with a quite sophisticated representation format (what is represented are the processes taking place in the world, not the patterns of information on which the perception of those processes is based).

- *Implicitness*. The motor simulation process (at least some of its aspects) can be partly accessed consciously (through a motor imagery episode), but most often it is executed in a purely non-conscious way. It is a subpersonal (Dennett, 1969) or subdoxastic cognitive process: it accounts for how we come to have certain beliefs about what we can do, but it is not itself accessible by introspection. Some ST advocates thus distinguish two types of motor imagery: motor imagery episodes which are accompanied by a conscious experience of the activity being simulated, and motor imagery episodes which take a purely implicit form (see e.g. Jeannerod & Frak, 1999; Jeannerod & Gallagher, 2002; Parsons & Fox, 1998). For the sake of precision, I think, however, that it is better to restrict the concept of imagery to mental activities involving awareness.
- *Realistic models of the body*. The internal models of the body used by the simulation mechanisms are constantly updated so as to maximize the correspondence between what is anticipated as feasible and what can really be done given the current state, skills, and resources of the body. These internal models make motor simulation sensitive to biomechanical constraints or velocity and time constraints characterizing the execution of real action, which is essential to build realistic representations. This is another difference with mental imagery, which might lack such sensitivity (Johnson, 2000). Undoubtedly, as demonstrated by several studies, some of the constraints on overt action are preserved in motor imagery, such as Fitt's law, or time and energetic constraints on body displacement (Decety & Jeannerod, 1995). Decety and Sommerville (2007) thus claim that ‘one reason why motor imagery allows us to plan actual actions is that the constraints of the physical world shape our imagery in a manner similar to how they shape our actions’. However, those observations do not demonstrate that mental – or motor – imagery *necessarily* complies with such constraints. After all, one can imagine oneself performing impossible actions, such as flying or lifting mountains, or actions which do not comply with the physical or biomechanical constraints of our body, e.g. stretching one's arm to catch distant objects (see Witt & Proffitt, 2008).
- *World-level representation format*. Motor simulation works with a sophisticated representation format which makes it possible to anticipate not only changes in sensory input (which is basically what sensorimotor emulation does), but concrete action possibilities and state of affairs.

All of these features are essential regarding the claim made by ST that motor simulation is responsible for our aptitude to perceive affordances, i.e. to anticipate what we can do with the objects in the environment. The implicit nature of the motor simulation mechanism fits with the way we experience affordances: you don't have to explicitly imagine yourself sitting on a chair to perceive it affords sitting; usually you take for granted this ‘sittability’ without even paying attention to it. The use of realistic body models and the capacity to take into account parameters relevant for real action execution is critical to represent feasible, not fanciful actions. Finally, the representation format is another key issue to explain affordance perception. If an information processing mechanism must be able to determine what can be done in the environment, it cannot merely emulate the sensory input changes that should be induced by a motor command (or sequence of motor commands), e.g. the changes in the optic array that this command should produce. As Cisek (2007, p. 1585) explains, ‘specification of actions […] requires information about the spatial relationships among objects and surfaces in the world, represented in a coordinate frame relative to the orientation and configuration of the animal's body’. To represent an action sequence such as grasping an object or using a wrench, the system must take into account its spatial and physical properties, for instance its position relative to the hand and the velocity of the arm during the different phases of the action, or the muscular effort that should be provided to master the inertial properties of the limb or the weight of the tool. A mere sensorimotor emulation of action sequences is not sufficient to predict action feasibility.

It would take too long to describe in detail the large corpus of empirical data which is generally considered as supporting ST. Without the claim of completeness and succinctly, the following observations can be mentioned (according to the distinction made above, the observations under points (a) and (b) concern mental imagery, whereas the observations under points (c) to (e) mainly refer to motor simulation):

- Mental chronometry measures demonstrate that the time taken to imagine an action corresponds to the time necessary for its actual realization and depends on the biomechanical and energy constraints associated with motor execution (Decety & Jeannerod, 1995; Decety, Jeannerod, & Prablanc, 1989; Frak, Paulignan, & Jeannerod, 2001; Gentili, Cahouet, Ballay, & Papaxanthis, 2004; Jeannerod & Frak, 1999; Parsons, 1994; Parsons & Fox, 1998).
- Neuroimaging data indicate that the performing of motor imagery tasks (e.g. categorization tasks involving mental rotation activities) is accompanied by the activation of most brain areas involved in actual movement execution (Decety, Jeannerod, Germain, & Pastene, 1991; Decety & Jeannerod, 1995; Jeannerod, 2001; Parsons, 1994; Parsons & Fox, 1998). The existence of interference effects when subjects rotate their hand while performing a hand-laterality judgment task also supports this idea (Sack, Lindner, & Linden, 2007; Wexler, Kosslyn, & Berthoz, 1998; Wohlschläger & Wohlschläger, 1998).
- The existence of stimulus-response compatibility effects in some categorization tasks suggests that visual information processing includes mechanisms of preparation to action, even when the subject is not engaged in a behavior involving the object (De'Sperati & Stucchi, 1997, 2000; Ellis & Tucker, 2000; Junghans, Evers, & De Ridder, 2013; Pappas & Mack, 2008; Tucker & Ellis, 1998, 2001, 2004; Wilf, Holmes, Schwartz, & Makin, 2013). In a task where subjects must judge whether objects in images are correctly oriented in the vertical axis, the responses are facilitated when they are given with the hand *most suitable* for grasping the object (Tucker & Ellis, 1998). Similar effects were observed for the orientation and size of objects and the type of hand grip or wrist rotation needed to align the hand for grasping the object (Ellis & Tucker, 2000; Tucker & Ellis, 2001, 2004). In addition, that similar effects are observed in patients with visual agnosia suggests that the mechanisms building the pragmatic representations supposedly involved in that case work at a fully preconscious and automatic level (Pappas & Mack, 2008).
- Studies on perceived reachability, with subjects exposed to a biased visual feedback of their motor performances, suggest that a process simulating the reaching action underlies the distance estimations and the functional delimitation between peripersonal and extrapersonal space (Coello, Bartolo, Amiri, Houdayer, & Derambure, 2008; Coello & Delevoye-Turrell, 2007; Delevoye-Turrell, Bartolo, & Coello, 2010). For instance, Coello and Delevoye-Turrell (2007) observed that a visual feedback manipulation giving the illusion of an extended arm reach leads the subjects to compensate with shorter movements and results in a phenomenon of constriction of the perceived reachable space.
- Distance estimation studies indicate that an experimental manipulation of the action capacities or behavioral potential influences the perceived egocentric distance (Proffitt, Stefanucci, Banton, & Epstein, 2003; Witt, 2011; Witt, Proffitt, & Epstein, 2004, 2005, 2010), but has no influence (i) if the subject expects to perform another action than the one affected by the manipulation (Witt et al., 2004, 2005, 2010) or (ii) performs a concurrent motor task during the distance estimation (Witt & Proffitt, 2008). The phenomenon of distance compression observed when a stick is used to reach targets (action performed immediately after the estimation) is neutralized if the subjects are instructed to manipulate a rubber ball while making the distance judgment, a simple squeezing action being sufficient to eliminate the effect. Conversely, subjects who merely anticipate or imagine holding a baton when they reach, estimate the targets to be closer than in the control condition. For Witt and Proffitt (2008), these observations suggest that a motor simulation mechanism is responsible for the calibration of perceptual representations on action capacities. Similar effects were observed for the estimation of hill slants (Bhalla & Proffitt, 1999; Proffitt et al., 1995, 2003) and size of objects (Linkenauger, Leyrer, Bülthoff, & Mohler, 2013; Linkenauger, Witt, & Proffitt, 2011; Stefanucci & Geuss, 2009; Witt, Linkenauger, Bakdash, & Proffitt, 2008; Witt & Proffitt, 2005).

## Why the ST's account of how we remember and perceive what can be done is not tenable

Taken together, the above elements can be considered as supporting two claims regarding the way action capacities are memorized: (a) the actions that the agent is able to perform are stored using internal models of the body and the environment, and (b) it is through motor simulation sequences that this memory becomes effective and is exploited to guide the behavior in the present, especially to build perceptual representations which are calibrated on the skills and capacities of the agent. This twofold thesis entails the two following empirical predictions: (i) a change in the action capacities of the agent which would not be reflected by an update of the internal models will not be taken into account in perceptual categorization;^2^ and vice versa: a misalignment of the internal models relative to the real capacities of the agent will result in the construction of incorrect perceptual representations; (ii) the perceived environment can only be scaled on the action capacities of the agent if a simulation mechanism making use of up-to-date internal models (that is to say, reflecting the actual state of capacity of the agent) is involved in the perceptual data processing system. In other words, the perceptual access to actions that are realizable in the environment at time *t* demands that the execution of these actions be simulated with up-to-date internal models and that the simulation process results in a positive output. As we shall see, however, several elements suggest that these claims and predictions are mistaken.^3^

### The affordances we are aware of at each moment are too many for their realization to be simulated (in parallel) by the brain

A first argument builds on the computational resources that would in principle be necessary if the brain had to simulate the actions supported by the objects to anticipate their realizability. In short, motor simulation is too costly computationally to explain how one can access prospectively to affordances through perception.

In ecological circumstances – typically in the rooms of a house – there is not one or several, but dozens or even hundreds of objects we perceive at every moment. More precisely, what we perceive is not a sum of objects, this is a whole articulated environment: the room or the house we are in with its familiar equipment. Certainly, our attentional focus is generally directed toward one single object or set of objects. We have nevertheless a peripheral perception of other surrounding objects. We are peripherally aware of their presence and availability. *And we implicitly know what they can be used for*; the field of possibilities which is made available by the environment is included in our awareness of the situation.^4^ In a way, such awareness even applies to objects that are *outside* our peripheral perceptual field, i.e. objects that do not appear but are nonetheless participating in the situation. I do not see the scissors lying on the desk behind my back. But I rely on their availability; the cutting action they enable belongs to my behavioral field. The same applies for any usable object located in our close environment, i.e. which is within range. We know that objects are available if we need them, and we know (more or less) where they are stored and how to find them.

Evidence for this claim is mainly phenomenological in nature. Relying on phenomenological data in the context of the present investigation is justified because what is at stake in this case is the *content* of our perceptual experience. We perceive more at each moment than the objects we are explicitly paying attention to. This point was especially made by Husserl: when we perceive an object, this object has an ‘external horizon’; the perception of the object is accompanied by a coperception of other present objects and to which it is possible to turn (see e.g. Husserl, 1950, §19).

We may have the impression that we are only aware of the portion of reality that is under attentional spotlight, but this is a mistake. Just as there is a central and a peripheral vision, our perceptual awareness is divided into a central and a peripheral field; we are constantly aware of the periphery, and this context or background contributes to determine the content of that which is the subject of our explicit perception. When I am visually aware of the coffee table in the living room, I do not perceive an object insulated from the rest of the world. The table is precisely perceived as being *in* the living room, which is in the house, which is in the city. The house, with all the objects – and related affordances – it contains, is, so to speak, coperceived when I see the table. The awareness – more or less implicit – of being located in a certain place of the network of familiar places the world consists of encompasses an awareness – more or less implicit – of the behavioral resources made available by that place. I know I am at the office, in a restaurant, in the subway, on the street, or at home in the living room. The ‘concept’ of living room as it occurs in my experience of being somewhere includes the objects this place generally contains, with the actions they potentiate, somewhat in the style of Marvin Minsky's frames.^5^

One may retort that strictly speaking we only perceive the affordances in the beam of our attention at time *t*; that such peripheral awareness is illusory and stems from our certainty of being able to turn our attention to the elements of the periphery.^6^ But in this case how do we explain that we know *where* to turn our attention? Most importantly, how do we explain that when planning our behavior we take into account opportunities for action provided by those structures that are not subject to direct perception? As Searle (2007) explains, the proof that such peripheral elements ‘are a part of my conscious field is that I can at any moment shift my attention to them. But in order for me to shift my attention to them, there must be something there which I was previously not paying attention to which I am now paying attention to’.

The work of E. Rietveld on selective responsiveness to affordances provides additional support to this view (see Rietveld, 2012a, 2012b; Rietveld et al., 2013). In order to be ‘selectively responsive to one affordance rather than another’, as we normally are, we must first be intentionally opened onto a whole ‘*field* of relevant affordances’ (Rietveld et al., 2013). This is especially the case for social affordances, i.e. possibilities for social interaction offered by the environment (Rietveld, 2012b, p. 208): ‘any relevant possibility for social interaction is embedded in a field of other soliciting possibilities for action. While engaged in a conversation with a friend, the cup of coffee on the table affords drinking from it, and my iPhone affords checking my email’ (Rietveld et al., 2013). But the same is true for ‘object affordances’. ‘It is the *whole* field of relevant affordances (social and other) that we are responsive to. This also explains why we switch so easily between interacting with a person and interacting with an object: we are immersed in an integrated field of relevant affordances, each of which can solicit activity’ (Rietveld et al., 2013).

These elements make quite obvious that motor simulation mechanisms *alone* cannot explain how one anticipates the action possibilities made available by surrounding objects. The affordances we are aware of at every moment are simply *too many* for their realization to be simulated by the brain.^7^ The neuropsychological and behavioral data available leaves no doubt: the number of actions that can be processed simultaneously by the motor system is very limited. The existence of interference effects when subjects have to execute overtly one action when running covertly another (Sack et al., 2007; Wexler et al., 1998; Witt & Proffitt, 2008; Wohlschläger & Wohlschläger, 1998) demonstrates that processing more than one action at the same moment is already beyond the computational capacities of the brain.^8^

This claim is reinforced when considering the ‘nested’ character of the affordances we access through perception. The action possibilities we anticipate when we perceive our environment are not atomistic pieces of meaning that could be considered in isolation from each other. They are organized as a network, where each possibility is related through conditional relations to others: the realizability of one given action is conditional on the realizability of others. Whenever one anticipates that an action A is realizable with an object O, one assumes that a set of other actions than A are achievable with O or other elements than O, and one makes a series of hypotheses about the behavior and properties that O or other elements than O will exhibit. To apprehend this glass across the room as an object that I can use to drink, I must anticipate that I can walk to the glass, that the floor is a solid surface capable of supporting my body, that I can reach out to the glass, that the glass is a physical object, not a hallucination, that it will not run away when I come near to it, that it is a solid object that will oppose resistance to my body, not a hologram, etc. One way or another, these assumptions are realized when I see that I can access the glass and use it to drink, even if, once again, I am only marginally aware of them.

Similarly, the perception of the action possibilities realizable in the environment obviously integrates an anticipation of the conditional and temporal constraints on action execution. Generally, using an object O affording an action A is only possible if other actions taking advantage of other objects than O are first performed in order to provide the conditions of realizability of A. As Wolverton (2011) explains: ‘An affordance A is actualizable by an effector E if and only if there is an irreducible sequence of affordances {A_1_,…, A_n_} in the environment such that for *i*={1,…, n-1}, A_i_+E → A_i+1_ and A_n_=A, where “+” signifies actualization and the arrow signifies an actualizable affordance A_i+1_ consequent to actualization of A_i_’. To borrow an example from the same author, a wine glass across the room will be perceived as ‘pick-up-able’^9^ only because the configuration of the environment affords a number of actions that must be executed before the glass becomes effectively ‘pick-up-able’. ‘The “pick-up-able” affordance offered by the glass […] is actualizable not immediately but via a sequence of perceived and actualizable affordances […]. The first (not really, but it will do) is the immediately actualizable affordance “stand-on-able” offered by the floor, etc., ending in actualizing the immediately actualizable affordance “grasp-able.” In this scenario are many affordances, not all necessarily perceived (but being dispositional, they are persistent). Among those that are perceived, the perceiver is always actualizing at least one (has to be supported by something). The others are not immediately actualizable, and therefore don't demand compulsory effecting. Some may be actualizable in the multiple-steps sense’ (Wolverton, 2011).

In this regard, the awareness we possess of what actions are possible in the environment (the affordances that are potentiated by objects) is organized according to similar principles as belief states, as described by Wittgenstein (1969), Quine (1951), Searle (1992), or Dreyfus (1992). The conditions satisfying the belief that object O affords action A (i.e. can be used to realize A) include beliefs which concern the realizability of other actions than A and possibly with other objects than O.

It should however be noted that in principle the above remarks, and the objection against ST which is based on them, are only admissible if: (*i*) we admit a holistic conception of beliefs such as the one proposed by Quine (1951). Quinean approaches to belief systems imply that belief evolution is not limited to isolated beliefs. Generally you cannot change your belief about A without also updating your belief about B and C. As Fodor explains, ‘the degree of confirmation assigned to any given hypothesis is sensitive to properties of the entire belief system’ (Fodor, 1983, p. 107); and if (ii) it is justified to equate our awareness of actions afforded by objects with *beliefs* about these actions. In terms of propositional attitudes, perceiving that object O makes possible action A means having the belief that P, where P is the proposition ‘it is possible to do A with O’. This approach can be challenged, especially when confronted with a first-person analysis of our ordinary experience of affordances. In ordinary perception, we obviously do not have to ‘believe’ (in the strong sense of a mental episode during which we are conscious to commit to the validity of P) that object O makes possible action A to behave in accordance with what we might call – for want of anything better – this ‘hypothesis’. Holding that O makes possible A means, at a more fundamental level than belief, to behave or be disposed to behave in conformity with this presumption. A striking illustration of this point is the experience we have of affordances related to objects’ solidity or impenetrability. I do not have to be engaged in an episode of belief where I ‘tell myself’ that a given object is graspable or that a surface is walkable to perceive them as such and behave accordingly. The simple act of stretching out my arm to hold on to a railing when slipping on a pavement testifies that I apprehend the railing as graspable and as likely to procure a stable support for restoring my balance. Surely, I can engage in a belief episode where I am explicitly aware (telling myself) that object O makes possible action A. However, this is not a condition for behaving in accordance with the presumption that O makes A possible.

Note that a dispositional account of belief (see e.g. Engel, 2005; Ryle, 1949) or an inferentialist approach to the conceptual content of beliefs such as the one defended by Brandom (2007) or Steiner (2014), can in principle deal with these phenomenological elements. To possess the belief that O makes possible A simply means to be disposed to behave in compliance with this belief in circumstances where this belief is likely to matter from a behavioral point of view.

### Affordances are not equivalent to currently or personally feasible actions

Another decisive objection against ST builds on the fact that most of the action possibilities we are aware of through perception are *intrinsic* (dispositional) properties of objects and *are not bound to immediate or even mediate realizability*. That is, they do not equate to (a) possibilities customized and referred to the specific agent who perceives them (what *he/she* can do); or (b) possibilities that are actualizable in present circumstances (what can be done *now*). The mechanism which is described by ST advocates is designed to evaluate the feasibility of a specified action given the current state, situation, and pragmatic resources of the individual, consequently it cannot account for the perception of this – ‘neutral’, in a sense – type of action possibilities.

The way ST proponents make use of the notions of ‘affordance’ or ‘action possibility’ is generally quite imprecise. Especially, they do not distinguish between actions that are possible ‘in principle’ with an object given its features for any referent agent having suitable body properties and skills, and actions that are feasible *right now* with this object, given the current state, position, and abilities of the perceiving agent and the current configuration of the environment. This is a problem because strictly speaking what an object affords at time *t* cannot be equated to actions that are realizable by the perceiving agent with the object at time *t*. Although relative to some characteristics and skills of a referent subject, what ecological theorists call his/her ‘effectivities’ (Turvey, 1992), affordances are not conditioned in their existence by the current realizability or the actions being afforded provided these effectivities. The resources that the environment offers exist – and can therefore be perceived – whether the conditions required for their exploitation by the individual are currently met or not. In short, affordances are not enslaved to immediate actualizability.

This is precisely where the problem lies when considering the ST account of affordance perception. ST advocates posit that perceptual access to affordances is enabled by a simulation process testing the feasibility of the afforded behavior, but a process assessing if the action of making use of an object is feasible in current circumstances cannot determine whether this object has or has not the related affordance. What the object affords does not depend on what can be done right now or in the immediate or far future (in other words the time step is of no concern). The only thing motor simulation can do is to anticipate whether a specified behavior taking advantage of a specified affordance will be a success or a failure given input parameters specifying current circumstances.

Supporting this view, recent studies have shown that the observation of graspable objects triggers activity in the motor cortex only when objects fall within the reaching space of the subject, suggesting that motor simulation is not involved for objects located too far away to be grasped (Cardellicchio, Sinigaglia, & Costantini, 2011; see also Costantini, Ambrosini, Scorolli, & Borghi, 2011; Costantini, Ambrosini, Tieri, Sinigaglia, & Committeri, 2010). These observations demonstrate that simulation cannot be responsible for the functional delimitation between peripersonal and extrapersonal space, contrary to what is suggested by Coello and Delevoye-Turrell (2007). If motor simulation is only triggered when an object falls in the reaching space, it means that other mechanisms than simulation are responsible for its delimitation.^10^

Similarly, using a task where participants were required to judge the laterality of graspable objects (images of cups with different orientations) presented alternatively in peripersonal or extrapersonal space, Horst, van Lier, and Steenbergen (2011) observed that object orientations for which the grasping action would be difficult because of biomechanical constraints were associated with increased reaction times only for stimuli presented in the peripersonal space. These observations suggest that the visual processing of objects automatically triggers the simulation of a grasping action when the objects are located in peripersonal space (at least, this is one possible explanation), but not when they are too far away to be grasped. Yet this is not because the object is beyond reach that it is not graspable and perceived as such. From the moment that the object has suitable properties – e.g. size, form, material – it affords grasping, whether within or beyond reach.

The same kind of remarks holds for the agent considered as the subjective referent of the affordances. Object O can be considered as affording action A for any agent capable of exploiting it, but the subject who perceives the affordance does not have, in principle, to fulfill these conditions. Authors such as Michaels (2003) even claim that the affordances we perceive do not have to possess any counterpart in our own behavioral abilities. More radically, affordances can be considered by reference to the effectivities of an agent which does not actually exist: for instance, air can be considered as graspable for aliens having air-grasping-prehensile-limbs. Of course, one major challenge such an approach to action possibilities must face is to determine what affordances a given agent in a given situation will tend to perceive, and how the perceptual system filters the actions afforded by objects to retain only the ones that are relevant. It seems reasonable to assume that most individuals will generally focus on affordances which are realistic considering their own situation, properties, and skills; but in no way does this mean that the possibilities we perceive are restricted to immediately actualizable actions.

The ability to anticipate actions which could be done with objects in other circumstances but cannot right now given current parameters, i.e. are not actualizable for the moment, also seems essential to explain heuristic behaviors (Michaels, 2003). To engage in a searching behavior aiming to set up the conditions necessary for the realization of a given action A – to borrow an example from Wolfgang Köhler's well-known studies on the insight phenomenon in monkeys (Köhler, 1925), finding something one can lean on to reach a for-the-moment-out-of-reach banana hanging from the ceiling – one must be capable of anticipating the actions that are possible *in principle* (provided that this and that conditions are fulfilled), but that are not *currently* feasible given our *current* position and resources or the *current* state of our body.

In addition, that (most of) the action possibilities we anticipate when perceiving objects are *intrinsic* possibilities, i.e. possibilities that those objects potentiate in principle, whether or not feasible in current circumstances, is demonstrated by several striking phenomena: (i) the fossilization phenomenon (see page 17), which leads to categorizing objects by reference to actions that can *no longer* be performed, whether because the instrumental means needed are no longer available or because the environmental conditions for their actualization are no longer (or not yet) fulfilled; (ii) the observation that the perceptual system reacts to virtual objects (e.g. pictures of objects) in the same way as it reacts to concrete objects (i.e. objects on which one can effectively act), by preparing the organism to actions directed toward these objects (see e.g. Pappas & Mack, 2008; Tucker & Ellis, 2001); and (iii) the intersubjective and social character of action possibilities that are anticipated when perceiving objects: in ordinary life, what an object is perceived to afford does not reduce to what one can do with it, it includes what others or a standard human being can do with it, or more generally what can *in principle* be done with it (for any agent capable of using it).

This latter point is perhaps most obvious when considering the role played by the anticipation of action possibilities in the categorization process, i.e. the process responsible for the assignation of identity, or more broadly, meaning to the objects we perceive, and the nature of the action possibilities which are anticipated in this process. As shown by Husserl (1907, 1913), in perception to identify an object O as an X (e.g. a solid object or, considering a lower level of abstraction, a table) always means to assume, presumptively, the realizability *de jure* of a set of possibilities with O. These possibilities are related either to: (a) the modalities of appearing of O, that is to say the types of experience of O we are likely to have in the further course of experience (seeing its other sides, touching its surface, etc.); (b) the types of processes in which O is likely to take part, i.e. what O is disposed to ‘do’ under this or that set of circumstances: O is disposed to break when struck, O is disposed to resist (not deform, not move) when a force is exerted on its surface, O is disposed to dissolve when immersed in a liquid, etc. (what is generally called the dispositional properties of objects); and (c) the types of actions that are realizable with O (for a referent agent): what O makes it possible to do, what we can do by exploiting the (dispositional) properties of O. The actions one anticipates as achievable when perceiving an object are also filtered by what is called a *naïve physics*. The concept of naïve physics refers to the prescientific and intuitive knowledge (which is partially sedimented in the know-how) that everyone has, of the laws governing the behavior of bodies and physical structures at our scale, and the way bodies are disposed to behave in different circumstances (Hayes, 1978). Our naïve physics plays an essential role in the identification of possibilities and dispositions that we spontaneously ascribe to the objects we perceive and it regulates, so to speak, our intelligence of situations. What we expect to be possible with objects is framed within the bounds of what we know to be the typical physical behavior of these objects. Note that this approach to categorization corresponds to what can be called an operationalist conception of the sense of the perceived object. The conditions of identity of the object are defined by the operations that can be performed with it: what the object makes it possible to do or more generally what the object can do, the behaviors it can exhibit, the roles it can play in a process, etc. In other words, once an object is identified, a commitment is made regarding the possibilities that this object, given its nature, is likely to actualize. What an object can do indicates what it is. And what an object is depends on what it can do (or what we can do with it).

The special nature of the possibilities which are anticipated in the categorization process is very well illustrated by the anticipation of unperceived aspects of objects. When we perceive an object visually, by definition we view it from a certain angle. Yet our experience of the object is not limited to the side we have before our eyes; it includes a presumptive representation of the sides that we cannot see for the moment, but that we *could* see if our position relative to the object was different. The possibility of seeing the other sides of the object is realizable in principle; it is a possibility *de jure*, which exists whether or not actualizable in the present circumstances (we may find ourselves immobilized). (A *de jure* possibility refers to something that is *necessarily* possible, that is to say, the proposition P stating that the possibility ρ is realizable with the object O is necessarily true: to make use of the possible worlds semantics, P is true in any (conceivable) world where O is likely to exist.) For Husserl (1907, 1913), this is this complex of expectations that enables us to apprehend the side currently before our eyes as the *front* of the object, and that enables us, more radically, to perceive spatial objects. Similar considerations apply to other categories of possibilities playing an equally vital role in our ordinary intelligence of reality. Impenetrability, for example, is an essential component of material objects. In any perception of material objects (i.e. ‘things’) it is assumed that it is not possible to pass through or occupy the area circumscribed by the object: we cannot *be* with our body where the object stands. These possibilities (and impossibilities) are what Husserl calls essential possibilities (Husserl, 1913, §.149). They are intrinsic constituents of the sense of the objects we perceive.

Once again, it seems obvious that our perceptual access to such possibilities cannot be explained by a data processing mechanism that would enable their anticipated realization, for reasons of computational resources on the one hand; but, on the other hand, and maybe more importantly, because this type of mechanism is designed to evaluate the feasibility of an action in current circumstances for the subject, not to determine what is feasible *in principle* with the object. The action possibilities contributing to defining the identity of the objects we perceive (what they are) correspond to actions that are realizable by definition with these objects, whether or not actualizable in current circumstances by the particular agent who is perceiving them. These possibilities cannot be equated to: (a) customized possibilities which are referred to the particular subject who is perceiving the object (what *he/she* can do given *his/her* current skills and situation) or (b) possibilities which are realizable in current circumstances (what can be done *right now*).

Distinguishing between these different types of possibilities is essential to determine what competence and empirical data the models being proposed in neuropsychology exactly explain, because the mechanisms supporting our awareness of each are assuredly different (though some of their subcomponents might overlap). For sake of clarity, I therefore propose to speak of: (i) *intrinsic possibilities*: action possibilities that are realizable in principle with an object for any referent system having suitable properties and skills; (ii) *immediately realizable possibilities* or ‘now actions’: actions that are immediately realizable at time *t* for a given agent; and (iii) *mediately realizable possibilities* or ‘after actions’: actions that are realizable at time *t* by an agent only if other actions are first realized. ‘Immediately’ here means ‘without any need to perform other actions or change something in the environment before’. The degree of immediacy being considered is of course arbitrary, in the sense that it is relative to a descriptive decision: one decides to consider that the cup in front of us is ‘immediately’ graspable, because we only have to stretch one's arm out to grasp it. Considering ‘stretching one's arm’ as one and the same action is what authorizes considering the grasping action as immediately realizable. Symmetric considerations apply for the meaning of ‘mediately’. Given an object affording action A, action possibilities of types (ii) and (iii) are always a subtype of (i). The definition of affordances originally given by Gibson is close to (i): affordances correspond to intrinsic action possibilities, not to now actions or after actions.^11^

## The direct content specification hypothesis

### The simulation theory's account of affordance perception suffers from circular reasoning

The arguments presented in the former section cast severe doubt on the capacity of ST to explain affordance perception. It must however be noted that these objections are only directed toward the claim, made by *some* of the ST advocates, that *any* action, in order to be anticipated as possible in perception, must be run covertly by the motor system.^12^ That is: the issue lies foremost in what *extension* must be assigned to ST. My view on this issue is that the explanatory significance of the motor simulation mechanism must be revised downwards. In addition, once the extension assigned to the motor simulation mechanism has been properly reduced, a major problem is to determine the prerequisite in terms of cognitive processing for this mechanism to work. This can be highlighted by the following point. Some authors such as Cisek (2007) propose to limit the scope of ST to actions that are already anticipated as realizable and that are potentiated by objects toward which our attention is focused: ‘It is not proposed that complete action plans are prepared for all of the possible actions that one might take at a given moment. First, only actions which are *currently* available are specified in this manner. Second, many possible actions are eliminated from processing by selective attention mechanisms which limit the sensory information that is transformed into representations of action’. However, as relevant as it may be, this limitation is far from being sufficient to solve the problem. Indeed, one must still explain how to determine for a given object (or situation or state of affairs) the actions (or, at another level of description, the motor programs) whose execution should be simulated. How can the system limit simulation to actions that are *currently available* if what actions from the behavioral repertoire are feasible in current circumstances is precisely what motor simulation must determine?^13^ In one way or another, a first layer of affordances must already be specified for the motor simulation mechanisms to work. In order for the brain to trigger a mechanism aiming at determining whether an object supports the realization of an action A_x_ from a behavioral repertoire {A_1_,…, A_n_}, for instance whether it can be grasped using a thumb-index precision grip, the object must *already* have been apprehended as belonging to the category of graspable objects, that is to say, the objects which afford *in principle* the achievement of this type of behavior (i.e. solid objects within a certain range of sizes). In short: the actions which are intrinsically realizable with objects must already be known by the system. Mechanisms other than motor simulation must consequently be hypothesized to explain how an assessment of action feasibility using motor simulation is possible.^14^

My position is that our knowledge of what can be done in the environment is supported by mechanisms enabling a direct specification from the structure of the *phenomenal content* of perception, that is to say, how things present themselves in experience (what analytic philosophy calls its representational content) conveys direct knowledge about what we can do. Visual distance perception is a good illustration of this mechanism. The visual gradient of distance enables us to locate objects in relation to our body, and assign to each a value in terms of accessibility. Through the gradient of distance, we are thus immediately aware of what can be done and what cannot. This is, so to say, written in black and white in the spatial layout we perceive. Now, psychologists have long shown that the configuration of this gradient (what is sometimes referred to as the perception of absolute distance) results from the – close to automatic – action of several monocular or binocular, static or dynamic optical variables (binocular disparity, height in the visual field, shadows and texture gradients of surfaces, relative sizes of objects, parallax, etc.), which together contribute to defining the position of objects in the distance (Cutting & Vishton, 1995). Let's mention two noteworthy rules: (a) for the sake of simplicity, consider a flat ground: if the point of contact of the base of an object A with the ground is higher in the optical field than an object B, then A is farther (in terms of egocentric distance) than B, and the additional distance is proportional to the height difference; and (b) if an object A masks an object B in the optical field, then it is closer. In addition, both rules are transitive: if A is higher in the optical field than B (or masks B) and B is higher than C (or masks C), then A is closer than C. This is true even if no direct comparison between A and C is possible based on optical height or masking. The conclusion is obvious: the mechanisms responsible for the construction of phenomenal distance enable a direct apprehension of the accessibility or inaccessibility of surrounding objects, without the need to simulate the action of accessing objects.

Another illustration is the visual perception of solid objects. When you visually perceive something as a solid object, e.g. a table in front of you, you take for granted that some behavioral possibilities are available (you can put objects or sit on its surface), whereas others are neutralized (you have to walk around the table to pass, you cannot be where the table is). Apprehending something as a solid object means precisely taking for granted such possibilities. In other words, one cannot *see* something as solid without *believing* that these possibilities are in principle realizable with the object. Here again, as in the case of distance, it is highly unlikely that the anticipation of such action possibilities and impossibilities proceeds from motor simulation mechanisms. What would be the point? As shown by Gibson (1958), the optical array is a reliable resource to specify solid surfaces. For instance, opacity almost always indicates tangibility. This is a highly robust regularity, which is only put into default in exceptional cases.

These elements can be systematized through what I call the *direct content specification hypothesis*. This hypothesis consists in claiming that, at least for the most basic actions of our behavioral repertoire (moving close to, passing through, striding over, standing on, climbing, reaching, grasping, pushing, pulling, lifting, carrying, throwing, etc.), the action possibilities we are aware of at time *t* through perception are directly specified by perceptual variables characterizing the content of our experience. That is, a given action A_x_ from our behavioral repertoire {A_1_,…, A_n_} is anticipated as realizable with a perceived object O based on the perceptual characteristics of this object in our experience: the way O appears (especially in connection with other objects and structures of the perceptual field) directly specifies (and ‘maps to’) A_x_. In other terms, the fact that this and that conditions are fulfilled in the perceptual field ‘activates’ (or ‘motivates’ if one prefers a phenomenological vocabulary) the belief that A_x_ is something (at least in principle) feasible, without the need to further process A_x_ through a motor simulation process or whatever. We know (or believe) that A_x_ is something that can be done with O, not because our brain has simulated the execution of A_x_ when processing the visual input, but because the phenomenal characteristics of O tell us so. Note that what specifies that A_x_ is afforded by O in this hypothesis is some property of the *phenomenal content* of experience, and not of the physical information (e.g. the ambient optic array) which is picked up by the perceptual apparatus. This precision must absolutely be kept in mind to avoid equating the direct content specification hypothesis with Gibson's theory of direct perception, but also to see why (and to what extent) both are complementary accounts in the overall task of explaining the perception of affordances. I will return to this issue below.

The direct content specification hypothesis thus claims that the cognitive system responsible for the perception of action possibilities is far more direct, in terms of cognitive processing, than what is stated by ST. Affordance perception makes use of shortcuts, so to say. Those shortcuts are essential to explain (a) how quickly the functional categorization of perceptual content works and how reactive we are to objects supporting actions, and (b) our ability to maintain a perceptual awareness of a whole field of action possibilities, i.e. our ability to perceive, even marginally, at one and the same time all that our environment affords. In comparison to such direct specification mechanisms, motor simulation appears slow and much more demanding in terms of computational resources. Because the motor system can hardly manage more than one action at a time (see page 7), it can only determine what is feasible in a sequential manner. In that regard, the direct content specification hypothesis is partly motivated by the assumption that cognitive processing obeys economical constraints.

### Filling in some blind spots of Gibson's direct theory of perception

The direct content specification hypothesis can be viewed as a phenomenological (i.e. first-person) complement to J.J. Gibson's direct theory of perception, which refers to the postulated relation of direct specification between the physical information available in the so-called ambient optic array (considering visual perception) and the affordances offered by the environment to the individual: that object O supports the realization of a given behavior can simply be extracted from the optic array structure, without further processing of visual data (Gibson, 1977, 1979). According to Gibson and authors such as M.T. Turvey, a direct perceptual access to affordances is made possible by: (a) the richness and precision of the stimulation furnished at the receptors (i.e. the energy distribution at the receptor surfaces), together with the sophistication and selectivity of the perceptual apparatus, which is able to extract from the stimulation, without further processing (in particular, without making inferences), patterns which directly *specify* typical affordances; and (b) the objective and ‘truly physical’ character of affordances: affordances are real macroscopic physical properties, similar to being liquid or solid for objects (Gibson, 1979; Turvey, 1974, 1992; Turvey & Shaw, 1979). Gibson relies on these claims to avoid an internalist account of meaning, for which the functional properties of objects are superimposed by the perceiving agent on a ‘functionally neutral’ physical reality and correspond ultimately to purely ‘subjective’ entities.^15^ Because affordances are like any other physical properties, they can be directly apprehended. The eye perceives them just as it perceives the position or size of objects.

However, such a realist account of affordances faces difficulties when it comes to explain how we are perceptually *aware* of affordances, i.e. of what can be done in the environment. That affordances are not subjective constructs, but fully real properties of the physical world, does not alter the fact that they only exist *potentially* when they are detected (which does not mean, of course, that they do not exist). When I perceive that a solid surface affords walking, I am not (yet) using it for walking. I could use it. But currently I am not. Therein lies the difficulty: How can mere possibilities be the subject of *direct* perception? Note that claiming that affordances are dispositional properties of fully actual structures, similar to properties such as the fragility of a glass (or the solubility or sweetening power of a piece of sugar), does not solve the difficulty, for except when one perceives that the glass is *currently breaking*, we can barely say that the fragility is something one directly perceives, something one *sees*: maybe this is something one knows, believes, anticipates, or takes for granted when perceiving the glass; but from a phenomenological standpoint, i.e. considering a first-person analysis of the intentional mechanisms providing access to such properties, it cannot be claimed that such properties are the object of *perceptual* awareness.

It is one of two things: either (i) the perceptual access to the environment is direct – i.e. not based on a representational mediation: the world is its own representation, as Brooks (1991) says – but then the claim that affordances are *perceived* appears untenable because the behavioral opportunities potentiated by the environment are only possibilities; or (ii) perception really allows to access (directly) to affordances, but then the question arises whether it can do without a representational mediation because only representation seems able to release perception from the physical world's actuality (see e.g. Clark & Toribio, 1994, p. 402, 412).

The information theory developed by Gibson and the concept of specification do not resolve the problem. That the ambient optic array has an informational structure which is sufficient to *specify* the behaviors that the structures of the environment potentiate (see e.g. Turvey, 1974) does not ensure a *perceptual access* to these behaviors. It must still be explained how mere possibilities come to be incorporated into the visual experience fed by the optical (or visuomotor) information or, at another level of description, are incorporated into the network of beliefs on which the individual relies to organize his/her activities. The visual field presents the structures and states of affairs that afford this or that behavior (a flat and solid surface that enables to walk), not the behaviors *per se* that are potentiated by these structures (the walking activity afforded by the surface). To say it another way: in order to be perceptually aware that something affords *doing action A*, in one way or another you must be aware of *action A* as something that is afforded by O. Action A must be part of the intentional content of your perceptual experience. One purpose of the direct content specification hypothesis is to address this issue.

To a certain extent, this objection against Gibson's account of affordances as ‘directly perceived’ is reminiscent of Fodor and Pylyshyn (1981)'s claim that the active extraction of invariants from the ambient optic array can at best enable the extraction of … optic invariants, precisely.^16^ The problem is: how does the perceiving agent ‘process’ the invariants that are being extracted so that they can acquire *an informative function*? How do the invariants extracted from the ambient light array come to be *about* ‘features of objects in the environment’, i.e. come to fulfill an intentional function, if not by an inference mechanism (Fodor & Pylyshyn, 1981, pp. 141–142)? The remarks I made above raise a similar issue (yet without endorsing a representationalist and inferentialist account of perception) but focusing on (a) the phenomenological content of perceptual experience, and (b) the counterfactual character of the afforded action which is supposedly the object of direct perception (as Gibson claims: one perceives *what objects afford*, i.e. the behavioral opportunity they support): how does the agent come to be perceptually aware of the action possibility *about which* the information is being extracted?

### Affordance perception from a first-person perspective

When explicitly deployed from a first-person perspective, the direct content specification hypothesis can be explained using the concepts of *sensational content*, *representational content*, and *belief content* (Crane, 1992, 2005 2009; Dretske, 1993; Peacocke, 1983).

As Peacocke (1983, p. 5) explains, ‘the representational content of a perceptual experience has to be given by a proposition, or set of propositions, which specify the way the experience represents the world to be’. That is, a perceptual experience has a representational content insofar as it presents objects, properties, facts, state of affairs, and so on, that are supposed to be the case in the environment. Perceptual experience is ontologically committed, so to say.

The *sensational* content of perception is more difficult to characterize, but can be defined negatively based on representational content as the ‘properties an experience has in virtue of some aspect – other than its representational content – of what it is like to have that experience’ (Peacocke, 1983, p. 5).^17^ One obvious way to isolate the sensational content of a perceptual sequence is to consider this experience while ignoring all that relates to the world as it is presented in that experience, i.e. its representational content. The sensational content of a given visual episode is what is left from this episode when you no more take into account the objects and state of affairs that are (re)presented by this experience (in general terms: the world). While standing completely still, you, for instance, consider what you see at time *t* as a bidimensional system of colored shapes, as a painter when he measures the sizes of objects in his visual field using his finger. That is, you neutralize the intentional function which ordinarily is automatically assumed by visual content, you do not treat any more what you see as something which is *about* the world (as if you took a picture of what you see and looked at it without paying attention to what it represents).

Finally, belief content can be defined as what you believe to be the case when you believe something. To put it simply, let's assume that beliefs have a propositional structure: that is, having a belief always means believing *that P*, and believing that P means assuming that the state of affairs described by P is the case in a reference universe (which is generally ‘the world’). Belief content is quite easy to define in this theoretical framework: when you believe *that P*, the content of your belief is simply P. To reuse the way Peacocke defines representational content: the content of a belief has to be given by a proposition, or set of propositions, which specifies the way the belief assumes the world to be. Beliefs are not necessarily related to perception, but in the present context we are only concerned with beliefs that are directly caused (or motivated, in phenomenological terms) by perceptual experience. Following the above characterization, perceptual belief content (belief content which is directly caused by perceptual experience) can be defined as what you believe to be the case when you perceive something, which once again can be described by a proposition or set of propositions.

Based on those distinctions, the direct content specification hypothesis can then be formulated as follows: the belief^18^ that one can perform action A with object O (or more generally given a particular state of affairs) is caused by (among other factors) a given representational content which is caused by (among other factors) a given sensational content or given functional relations (e.g. relations of covariation) between elements of sensational content. For example, the belief that this glass is within arm reach (i.e. that it can be reached with a simple arm movement) is caused by a given configuration of the optical field. No motor simulation shall intervene in the processing of visual data. The configuration of the optical field (i.e. sensational content) determines the representational content: ‘the glass at this distance in front of me’, which determines the belief that the glass is at reaching distance.^19^ As such, this mechanism may seem simplistic. Its strength does not lie in the associations it performs, which are, as the name implies, direct, but in the route that led to their establishment, i.e. in the history of the genesis of these associations. Going back to the case of reachability perception, such simple associative mechanisms work because past experiences of reaching with the arm have enabled the calibration of the optical field on reaching capabilities, i.e. on the operational distance covered by arm reaching. This is precisely how ‘action capacity memory’ works in this case.

Once again, I must emphasize that this account of affordance perception is not identical to the theory of direct perception initially proposed by Gibson, and later refined by authors such as Turvey, Shaw, Reed, and Mace (see especially Turvey, 1992; Turvey & Shaw, 1979; Turvey, Shaw, Reed, & Mace, 1981). At least two elements can be put forward to delimit both accounts, in their explanatory scope as well as in the explanatory apparatus they make use of:

1. As stressed before, the focus of the direct content specification hypothesis is phenomenological: what is under scrutiny and is to be explained is the *content* of our perceptual awareness. In contrast, Gibson's theory of perception is (mainly^20^) a third-person approach to perception: it aims to explain how a living system can extract from the physical information structures available (e.g. the ambient light) patterns specifying behaviorally relevant properties (i.e. properties of the animal-environment system). Gibson's theory explicitly endorses a physicalist perspective and its explanatory scope remains behavioral. In addition, from the perspective promoted by the direct content specification hypothesis, the content of perceptual experience can be considered as playing a causal role in behavior, and thus as something which, to a certain extent, explains why the subject exhibits this or that behavior.
2. An additional difference is – to build on the remarks made on page 13 – that the direct content specification hypothesis directly addresses the issue of how the behavioral possibilities anticipated as realizable (e.g. the possibility of reaching and grasping this object or the possibility of climbing these stairs) come to participate in perceptual awareness: what is their intentional status. In that regard, I do not claim with Turvey (1994) ‘that the *behavioral possibilities* of surface layouts and events [are] *perceived*’ (emphasis added), because I think that their intentional status in perceptual awareness is different from the one of the structures that potentiate (i.e. afford) these behavioral possibilities. Strictly speaking, behavioral possibilities are not something I *see*: I see that the chair affords sitting, or I see (or treat) the chair as something that affords sitting; but I do not see the sitting behavior which is afforded by the chair (I would see it if I saw John actually using the chair to sit). That's why I make use of the concept of belief, but indicate in addition that those beliefs do not have to take an explicit form when the perceptual episode is occurring (I do not have to ‘tell myself’ that I could use this chair to sit when, perceiving the chair, I come to believe that it is sittable).

Those differences do not imply that the direct content specification hypothesis and Gibson's direct theory of perception are incompatible explanations of affordance perception. They rather constitute complementary accounts: the process of extraction of patterns of physical information specifying affordances, to which perception is equated in Gibson's account, is a possible third-person counterpart of the mechanism of content specification, which is postulated by the direct content specification hypothesis as subtending the intentional access to affordances in perception. Note however that my objective in this article is not to propose a fully blown account combining first-person and third-person aspects of affordance perception. Additional work needs to be done to describe more precisely how those perspectives must be articulated. I will only mention that a possible epistemological framework to combine both accounts is *anomalous monism* (Davidson, 1970), which is sometimes credited to Husserl (see Smith, 1994). Anomalous monism considers that first-person and third-person accounts are distinct possible descriptions of the same phenomena. Both accounts address the same reality, and may be complementary considering the overall objective of explaining ‘how the mind works’: both shed a different light on the same process, they address different dimensions of the *explanandum*. One important specificity of anomalous monism, however, when compared to other forms of monism, is to refuse any laws (e.g. causal laws) that connect physical and phenomenological descriptions of events (see Smith, 1994, p. 159).

Now that this clarification has been made, let me return to the mechanisms enabling a direct specification of perceptual content. A basic mechanism that can be used to implement such direct mapping relies on retinal sizes of objects. The surface occupied by objects in the optical field is a quite robust hint to specify their egocentric distance (at least for familiar objects), and such distance is probably (at least partly) defined in operational terms, e.g. in terms of reaching possibilities. From a computational point of view it is quite simple to associate for a given object, say a glass, a set of values in terms of optical surface being occupied with a reachability limit. Such direct mapping enables us to categorize quite directly retinal sizes on reaching capacities.

Of course, the optical field, as such, is not sufficient to convey knowledge of what can be done in the environment. As Rochat, Goubet, and Senders (1999) explain, ‘reachability is codetermined by the characteristics of the object and those of the actor in terms of his/her capacity for action and situation in the environment’. That is, the calibration of optical distance on reaching capacity is necessary. However, once the optical cues have been calibrated on reachability performances enabled by the arm, they suffice by themselves to specify if a target is reachable (see e.g. Carello, Grosofsky, Reichel, Solomon, & Turvey, 1989; Fajen, 2005; Fajen, Riley, & Turvey, 2009; Gabbard & Ammar, 2005). In other words, our body can serve as a metric to calibrate the optical field without the mediation of motor simulation (see e.g. Mark, 1987; Warren & Whang, 1987). For reachability, the only requisite is to memorize the reach of our repeated arm movements using optical cues, i.e. to use the grasping distance *as defined in the optical field* as a metric to calibrate visual distances. The same mechanism could be used to calibrate the size of objects using the metric of our hand grip span (Linkenauger et al., 2013).

The phenomenon of egocentric distances compression which is observed when subjects use a tool extending their arm reach (Berti & Frassinetti, 2000; Longo & Lourenco, 2006; Witt, 2011; Witt et al., 2005, 2010), or are provided with a biased visual feedback of their reaching performances making them believe in such extension (Coello & Delevoye-Turrell, 2007), also supports this hypothesis. What is demonstrated by these observations is not that the perception of objects’ reachability relies on motor simulation mechanisms testing covertly the feasibility of the reaching behavior, but that the visual reachability space is calibrated on (what we visually perceive of) our action capacities, and that (what we visually perceive of) these capacities play a metric role in the determination of egocentric distances. Perceiving that we are able to reach farther in visual depth causes a compression of the apparent distance of objects: the same object which looked farther now looks closer. What is postulated by the direct content specification hypothesis is that such reconfiguration can be described as a modification of the functional relation between sensational content and representational content: after the reorganization of egocentric distances caused by tool use, the same configuration of the optical field motivates a different representational content (the spatial layout of objects – in that specific case: their egocentric distance – has changed), which in turn motivates new beliefs about what one can do (which objects are reachable, which objects are not).

Another empirical support to the direct content specification hypothesis comes from studies on force perception. When one grasps an object and tries to lift it, the object appears more or less heavy. It has been repeatedly demonstrated that the perceived heaviness is based on the perceived effort (i.e. how hard one tries) required to move the object, which in turn depends on the maximum weight the subject is capable of lifting (Bertrand, Mercier, Shun, Bourbonnais, & Desrosiers, 2004; Cafarelli, 1988; Cafarelli & Layton-Wood, 1986; Carson, Riek, & Shahbazpour, 2002; Gandevia & McCloskey, 1977; Jones & Hunter, 1983a, b; Simon, Kelly, & Ferris, 2009). The perceived heaviness is a ratio between the absolute heaviness of the object and the maximum force the subject is capable of producing, i.e. the force he would develop as a result of the maximum effort he is capable of. The more important the proportion of the available force required for lifting the object is, the more it seems heavy. Perceived heaviness can consequently be considered as a perceptual content mediating knowledge of our lifting capabilities, and as a kind of affordances. Through perceived heaviness we anticipate what we can do with the object and at what cost. Light objects are objects that can be (easily) lifted and handled. Heavy objects are not: either they cannot at all be handled, or they can only be handled at the cost of an important effort. The question is: how is perceived heaviness determined? According to ST, in order to determine if an action is feasible with an object, one must simulate the action of using this object based on internal models of the body capabilities and of the behavior of the environment. Does the available data support such view? Clearly not. The calibration of perceived heaviness on the force the subject is able to produce is based on purely embedded mechanisms: (a) the perceived effort depends on (i.e. is psychophysically related to) the degree of activity in the motor areas generating the muscular command, and (b) the activation of the muscles requires a motor command more important when it is close to the maximum force they can produce; as a result, (c) the perceived effort will be more important when the force produced is close to the maximum force the muscles can develop. This purely embedded mechanism does not require any motor simulation process. In addition, one key point for the direct content specification hypothesis is that our awareness of what we can do when manipulating an object is entirely *implicated* in the object's apparent heaviness. That is: our awareness of what we can do with the object given our muscular resources is nothing else that our awareness of its weight. Weight is exactly like apparent distance, it is a way we are aware of what we can do through immediate perceptual awareness.

### The fossilization phenomenon

A more indirect but equally important empirical support to the direct content specification hypothesis which deserves to be mentioned comes from observations suggesting the intervention of what we call a phenomenon of *fossilization* of affordances.^21^ Fossilization can be defined as a gap between the capacities that are treated as available by the cognitive system and the capacities this system really has at its disposal. This can be described in the following terms. Ecological psychologists following Gibson often describe affordances as relational properties (Stoffregen, 2003; Turvey, 1992): affordances are properties of the environment taken by reference to properties or skills of the agent, what is called his ‘effectivities’ (see page 8). In these terms, the fossilization phenomenon refers to a situation where the affordances being perceived refer to effectivities which have no more ‘effectivity’ in the behavioral repertoire, skills, or characteristics of the agent. A fossilized affordance can thus be defined as follows: an object O (or any state of affairs) is apprehended by the subject S as potentiating an action A (S perceives that O affords A or, more generally, treats O as potentiating A), while the conditions, considering the effectivities of S, to exploit O so as to realize A are no longer fulfilled.

A striking example of fossilization is provided by some observations made by Iriki, Tanaka, and Iwamura (1996) and Iriki, Tanaka, Obayashi, and Iwamura (2001) in their well-known studies on the influence of tool use on the visual receptive field (RF) of bimodal neurons in macaques. The authors measured the activity of two kinds of bimodal neurons: (1) proximal-type neurons reacting to visual and somatosensory stimuli confined to the hand, and (2) distal-type neurons reacting to somatosensory stimuli located in the area of the shoulder and the neck and to visual stimuli in the space reachable with the hand. They observed that the repeated use of a rake by macaques to grasp objects beyond manual reach caused: (a) for proximal-type neurons, an expansion of the visual RF from the hand to the extremity of the tool, such that the neurons now reacted to stimuli near the extremity of the rake; and (b) for distal-type neurons, an expansion of the visual RF to the areas that could be reached with the tool. After a learning period of several weeks, a few minutes of tool use were sufficient to cause this reconfiguration. The authors also observed that the expansion only occurred when the macaque was actively using the rake; a passive grasp of the tool had no effect. A number of psychologists have suggested, on the basis of these observations and others,^22^ (i) that using a tool to reach objects causes an extension of the peripersonal space and (ii) that this extension could be the result of the integration of the tool within the body schema: the tool is treated as an extension of the hand, thus resulting in an extended reachability space.

What is noticeable for the present discussion is that Iriki et al. (1996) observed that following extensive tool use the extension of the visual RV of the bimodal neurons persisted several minutes after the tool was left. This observation suggests that during this time period, the brain was still treating some areas of the surrounding space as reachable although these areas could not be reached any more, i.e. the agent was seeing the environment as if he could still act in it with the power of the tool although this power was no more available. This is what we call *fossilization*. In a sense, fossilization is a consequence of a lack of reactivity or plasticity of the action-perception system. Such a loss of plasticity can be problematic in some situations because it can engage the individual in behavioral decisions which are in fact beyond his capacities, but it has also advantages: fossilization is a way of embedding what is statistically normal or what is generally true, and this looks like a clever mechanism to spare computational resources.

The fossilization mechanism can in principle be generalized to any kind of action possibilities (whether or not supported by instrumental means), considering that most actions of our behavioral repertoire can be actualized only if precise conditions are fulfilled and particular resources are available. In the case highlighted by Iriki et al. (1996), the fossilization phenomenon is reversible and restricted to a very short period (a few minutes). But this is not always the case. Paralysis, understood as the unavailability of the whole (or almost the whole) motor possibilities repertoire, provides a striking example of fossilization, because in this case the subject continues to see the world in terms which in a way or another presuppose the availability of this repertoire. Although he can no longer move to the objects, the paralytic still perceives a gradient of distance which is calibrated on his lost movement ability. Far objects still appear less immediately accessible, i.e. further away, precisely, than nearby objects.

Amputees suffering from phantom limb syndrome provide a similar example of fossilization. It is quite frequent to see amputees still relying upon the availability of their missing limb in their motor behavior, thus demonstrating a kind of functional blindness to the loss of their limb. Some leg amputee persons sometimes try to make use of their phantom leg to walk, then explaining that they have momentarily forgotten its absence (see the cases reported by Gallagher & Meltzoff, 1996; see also Gallagher, 2000). Likewise, Poeck (1964) reports the case of a woman who lost her thumb when she was five, and still tries to make use of the missing finger when trying to grasp objects 50 years later. ‘Every time she handles an object with her right hand, she tries to grasp it as if the missing member were still present. Even today, it is only when her grip fails that she becomes consciously aware of her defect’ (Poeck, 1964, p. 272). As Gallagher and Meltzoff (1996, p. 7) explain, this kind of oversight suggests ‘that the missing limb continues for a time to function schematically in a normal way in motor behavior’, its absence is not taken into account. The repertoire of action possibilities potentiated by the effective availability of the limb is fossilized, just as the affordances which are perceived as their counterpart on the side of the environment. The fact that the Simon effect in peripersonal space remains unaffected by limb amputation – i.e. unilateral amputees still show an advantage in response speed when visual stimulus and response correspond spatially, regardless of whether the stimuli are located in the hemispace ipsi- or contralateral to their missing limbs – also supports this interpretation (Philip & Frey, 2013).

These observations tend to demonstrate that fossilization is one essential mechanism involved in the capacity we have to keep track of what we can do. In addition, they provide indirect support to the existence of the direct content specification mechanism described above. Of course, in principle ST can also explain the data accounting for fossilization. One can typically claim that in these circumstances what is ‘fossilized’ is the internal models storing the action capacities of the body. A mismatch between the (fossilized) internal models used by the motor simulation system to evaluate what is feasible and the effective body capacities of the subject would explain that something which in fact cannot be done is perceived as afforded by the environment. However, this explanation seems quite unlikely: (i) the advantage of simulation is precisely meant to be its reactivity and ‘up-to-dateness’ regarding the effective status of the individual (see e.g. Decety & Sommerville, 2007); and (ii) the effects observed in the studies cited above, especially in Iriki et al. (1996, 2001), do not seem to engage the motor system. The effect of a visual object on the bimodal neurons whose visual RF is extended to include the tool is ‘direct’, in the sense that it does not appear to be mediated by any information processing mechanism aiming to evaluate if the object is or not reachable. On the contrary, it is probably *because* bimodal neurons appear to be activated by the visual input of this or that object in the peripersonal space that the cognitive system treats the object as located at reaching distance.

## Conclusion

Taken together, the arguments proposed in this paper demonstrate (i) that the claim that perceptual access to affordances is mediated by an action simulation mechanism rests on a misunderstanding of what affordances are and is anyway confronted with a computational reality principle and (ii) that the very functioning of the motor simulation mechanism, as it is described by ST, already presupposes some perceptual knowledge of the action possibilities made available by the environment. This implies a drastic reduction of the explanatory significance that can be attributed to the motor simulation mechanisms when considering the problem of what cognitive processes enable human beings to gain perceptual knowledge of what they can do based on the memory they keep of their past performances.

These considerations do not mean that motor simulation cannot be part of the cognitive processes responsible for the perceptual knowledge of what we can do. However, *when* precisely motor simulation plays a role and *what* it is *for* exactly remain largely unknown currently. One hypothesis is that motor simulation only intervenes when other, more direct, action feasibility estimation mechanisms lose their reliability, e.g. when an object to be grasped is located at the boundary of the arm reachable space. Supporting this view, Coello et al. (2008) have shown that transcranial magnetic stimulation of the motor cortex only interferes with judgments of reachability for targets located at the limits of reachable space, which tends to demonstrate ‘that action simulation would be required mainly when the determination of what is reachable becomes ambiguous’. The fact that the time required for judgments increases substantially for visual targets located in this critical area further supports this hypothesis (Bourgeois, Bartolo, & Coello, 2009). Another hypothesis is that motor simulation might come into play when the realizability of a complex motor plan needs to be evaluated, for instance when an object to be reached is behind an obstacle (see e.g. Morgado, Gentaz, Guinet, Osiurak, & Palluel-Germain, 2013), or when complex hand rotation and limb orientation are necessary for grasping an object (such as in the protocol of Frak et al., 2001).

It shall also be noted that neuropsychological data demonstrating that the visual perception of an object automatically triggers some activity in the motor areas (especially when the object is located in the peripersonal space) does not necessarily mean that a data processing mechanism is *simulating* an action directed to the object with the purpose of determining its feasibility in current circumstances, i.e. ‘provide the self with information on the feasibility of action potentials’ (Coello & Delevoye-Turrell, 2007). Another interpretation of these observations is that the body is preparing for the eventuality of having to interact with the target: motor circuits are activated not to execute covertly the motor plan, but to be more responsive and efficient in case of effective action (see Wilf et al., 2013, for empirical support to this view). And it is precisely because the target has already been categorized as located within the reachability space that motor circuits are found to be activated in that case. It's like making the engine roar when waiting at a red light. Of course, this alternative hypothesis raises other difficulties. In particular, it requires that we determine what it means, exactly, from a neurocomputational point of view, for the motor circuit to ‘prepare’ that way, and why such a ‘preparation’ is likely to improve responsiveness and efficiency. However, this explanation is at least as plausible as the explanation proposed by ST.

Finally, one limit of the mechanisms postulated by the direct content specification hypothesis is that they presumably apply only to basic actions of our behavioral repertoire. That's why the direct content specification hypothesis is more complementary than alternative to ST. Anticipating the realizability of more complex actions (e.g. actions combining complex gestural sequences) probably relies on other mechanisms. Typically, evaluating how a device should be operated, if a piece should be rotated or pulled, or how to place one's hand on it, probably requires motor simulation or even motor imagery: we have to imagine ourselves using the device to understand how it works or determine whether we can action it given our current posture and location in space. This also implies that motor simulation might primarily be used not to determine *what* can be done or *whether* it can be done, but *how* it can be done: what sequence of elementary actions must be performed, how the sequence should be organized (e.g. which action comes first: pulling or rotating). This hypothesis is compliant with at least some of the empirical data available. For example, the observations made by Ellis and Tucker (see page 5) show that “*how*-components” of actions are potentiated during the visual processing of the object, but they do not support the hypothesis that motor simulation mechanisms are involved to determine *whether* a given action is or is not feasible. That a precision grip can be executed is generally immediately evident when one sees the object. How this precision grip can and should be performed given our current body position, and the shape and orientation of the object, is another story.

## Footnotes

1 The simulation model is used to account for many other cognitive abilities, especially the ability to attribute mental states to others (mind reading) or, more radically, to understand others as intentional agents. These works will not be discussed here.

## Footnotes

2 As Witt and Proffitt (2008) explain, ‘the outcome of the simulation, rather than the perceiver's ability at the time of estimating distance, influences perception’.

## Footnotes

3 I have presented some of these arguments in another article (Declerck, 2013). To avoid building from scratch, I partly draw on them in the present paper.

## Footnotes

4 A negative support to this claim can be found in the spatial disorientation phenomenon. When you are disoriented – you are somewhere but you are incapable of determining where you are, e.g. in the immediate period following an accident – what happens is precisely that this field of possibilities is erased from your awareness of the situation.

## Footnotes

5 Based on Heidegger's phenomenological analysis of our everyday coping with objects, Turner (2005) makes a similar claim: affordances participate in a network and we never perceive them in isolation.

## Footnotes

6 A similar argument was used by O'Regan and Noë (2001) against internalist approaches to perception.

## Footnotes

7 An additional point which further undermines the computational credibility of ST is related to what can be called negative affordances. One can perceive objects as – more or less immediately – reachable, but obviously one can also perceive them as out of reach, i.e. as *not* reachable given one's current respective position and action resources. If, as claimed by ST advocates, motor simulation mechanisms explain the calibration of visual distances on the reaching or moving-close-to capabilities of the perceiving agent, does it mean that to register a visual object as out-of-reach one must run a simulation of the reaching action and this simulation must end with a negative output? That is to say, is the perception of negative affordances the outcome of a failing simulation? That seems highly improbable.

## Footnotes

8 It is worth noting, however, that these data deal with the capacity to perform a given action while engaging into the explicit (i.e. motor imagery) or implicit simulation of another action, and that, currently, there seems to be no data demonstrating that the brain cannot manage several actions simultaneously when processing them *only covertly and in a purely non-conscious way*. It is a widespread observation that in many cognitive tasks performances are better when the task is achieved without (or with low) conscious monitoring (see e.g. Rousselet, Fabre-Thorpe, & Thorpe, 2002, for the demonstration that subjects are able to process in parallel several images presented simultaneously so as to extract information about high-level (i.e. semantic) objects properties). One could thus expect better performances for purely implicit motor simulation, than for motor imagery or overt action execution, or a combination of both. This issue needs to be investigated, but, whatever will be found, it will not destroy my argument. For even if it turns out that the motor system is able to process simultaneously more actions than we suppose, it is highly unlikely that it can manage the (huge) number of action possibilities we are at each instant aware of–though implicitly and peripherally (see page 6). The claim that each affordance we are aware of results from a corresponding ongoing motor simulation process remains in any case implausible. I am grateful to an anonymous referee for drawing my attention to this issue and empirical data.

## Footnotes

9 More precisely: the action of ‘picking-up’ the glass will be anticipated as realizable. As I will explain below, perceiving that an object offers an affordance such as the pick-up-ability must not be confused with perceiving (or anticipating) that the action is realizable (i.e. actualizable in the current circumstances). The glass remains ‘pick-up-able’ even if I cannot pick it up *right now* and it remains ‘pick-up-able’ even if *I* cannot pick it up (maybe others can).

## Footnotes

10 Note that the overcapacity argument presented on page 6 is directed toward the claim made by ST (at least by some of its advocates) that one's perceptual access to affordances is *always* subtended by motor simulation processes, whether or not the object falls within one's peripersonal space. To that extent, these empirical data do not weaken the argument.

## Footnotes

11 The claim that affordances are not enslaved to immediate actualizability probably does not apply equally well to all kinds of so-called affordances. For instance, it could be stated that reachability necessarily refers to the possibility of reaching the objects *here and now*, given the relative position of our body and the reach of our arm. Current exploitability would be in that case a constitutive component of the affordance. But this is clearly not the case for other types of affordances: a given solid surface affords support for standing and walking even if I am not in position to use it or – more radically – even if I am, for any reason, *unable* to use it (I might be paralyzed). That's one reason why the distinctions proposed here are important, whereas the notion of affordance, as it is generally used, is probably too general to be relevant. The notion of reachability only applies to the action possibilities of types (ii) and (iii). No real object is *in principle* reachable (or all objects are). Solid visible objects are reachable when certain proximity conditions are fulfilled. It means that if we accept that the notion of affordance only applies to action possibilities of type (i), reachability *is not* an affordance.

## Footnotes

12 For instance, Garbarini and Adenzato (2004) explain: ‘only by virtually executing the action can we understand the relational significance of the object, i.e. the affordance it offers’.

## Footnotes

13 This issue recalls a well-known objection to the frame approach in artificial intelligence: if the knowledge the machine has of its situation amounts to a set of descriptive propositions (declarative knowledge stored in a base of facts), the problem arises of how to determine which propositions must be reevaluated when a change occurs in the environment or in the situation of the machine, e.g. its position. As Fodor explains: ‘How […] does the machine's program determine which beliefs the robot ought to reevaluate given that it has embarked upon some or other course of action?’ (Fodor, 1983, p. 114).

## Footnotes

14 Exactly the same objection can be made against the following claim of Gallese (2000): ‘To observe objects is therefore equivalent to automatically evoking the most suitable motor program required to interact with them. Looking at objects means to unconsciously “simulate” a potential action. In other words, the object representation is transiently integrated with the action-simulation (the ongoing simulation of the potential action)’. How does the cognitive system know in advance what are the ‘most suitable motor programs required to interact with [the objects]’?

## Footnotes

15 See especially Reed (1988, p. 231) who quotes a handwritten note from Gibson where this idea is expressed with particular clarity.

## Footnotes

16 ‘Although he never discusses the issues in quite these terms, it is reasonably evident from Gibson's practice that he wishes to distinguish between what is *picked up* and what is *directly perceived*. In fact, Gibson ultimately accepts something like our first constraint – that what is picked up in visual perception is only certain properties of the ambient light array. Gibson is thus faced with the problem of how, if not by inferential mediation, the pickup of such properties of light could lead to perceptual knowledge of properties of the environment. That is: how, if not by inference, do you get from what you pick up about the light to what you perceive about the environmental object that the light is coming from?’ (Fodor & Pylyshyn, 1981, p. 143).

## Footnotes

17 This characterization of sensational content, because of its purely negative character, is not very satisfying: it tells what sensational content *is not*, but it does not tell what sensational content *is*. A more acceptable definition would build on Husserl's characterization of experience as organized through layers. What is sensational for this approach can be specified based on a series of ‘reductions’, i.e. abstraction operations. See e.g. Husserl (1907, §. 14 and §. 15).

## Footnotes

18 For sake of simplicity, we assume that our knowledge of what can be done in the environment has a belief format (see page 8). More exactly, this knowledge is the object of dispositional beliefs which are automatically caused by perception: when perceiving an object O, we know what we can do with (or given) O insofar as we possess the belief that actions {A_1_,…, A_n_} are possible with O.

## Footnotes

19 Note that this formulation of the direct content specification hypothesis does not entail a commitment to representationalism, in the classical sense of assuming the existence of neural states that stand in for state of affairs in the external world (see e.g. Clark & Toribio, 1994; Degenaar & Myin, 2014). Most accounts of perception assuming a form of representationalism make use of the concept of content and most anti-representationalist accounts build on an attack on this concept (see e.g. Hutto & Myin, 2013). However, the concept of content, and especially of *representational content*, which is used here is a purely *phenomenological* concept: it is not stated that neural states (or any part of the organism of the perceiving agent) represent properties of the external world; it is stated that perceptual awareness, understood as an intentional state, is something that presents the world to be in this or that way. Or, to say it a bit differently, in order to describe the perceptual experience of a subject, you need to take into account that this experience presents the world as being this or that way.

## Footnotes

20 Whether and to what extent Gibson's direct theory of perception can (also) be considered as a first-person theory of perception might be discussed. My position is that Gibson's theory is better interpreted as a purely third-person account of perception. Undoubtedly, many of Gibson's claims seem to have some phenomenological significance. However, these descriptions are not elaborated in a systematic and methodologically sound way: Gibson does not rely on a specific descriptive method or make use of a dedicated conceptual system; to a large extent, the ‘first-person’ descriptions he proposes remain ‘naïve’, in this sense. In addition, perception is not analyzed and studied by Gibson as a conscious state with phenomenological properties, but as a behavioral category: some behavioral patterns are interpreted as patterns of ‘perceptual activity’. As Turvey (1974, p. 166) explains, for Gibson, ‘perception of the environment corresponds simply and solely to detection of […] variables of stimulation’. Consequently, Gibson's theory simply *does not need* to include a first-person account of perceptual experience to be a theory of perception. To explain how perception is possible, it is sufficient to explain how the detection of these variables can occur.

## Footnotes

21 The principle of the fossilization phenomenon has been presented in Declerck and Gapenne (2009) and Declerck (2012) on the basis of neuropsychological and phenomenological data.

## Footnotes

22 The observations of Iriki et al. have been replicated in humans by studies involving line bisection tasks with healthy subjects (Longo & Lourenco, 2006, 2007) and subjects suffering from neglect (Berti & Frassinetti, 2000; Cowey, Small, & Ellis, 1994; Halligan & Marshall, 1991; Pegna et al., 2001), distance estimation tasks (Witt & Proffitt, 2008; Witt et al., 2005), intermodal extinction (Farnè, Iriki, & Làdavas, 2005; Farnè & Ládavas, 2000; Maravita, Husain, Clarke, and Driver, 2001), and intermodal interference (Maravita, Spence, & Driver, 2003; Maravita, Spence, Kennett, & Driver, 2002; Maravita, Spence, Sergent, & Driver, 2002). Several studies also corroborate the observation made by Iriki et al. (1996) that the active use of the tool is necessary for the phenomenon of reconfiguration of body schema and peripersonal space to occur (see e.g. Farnè et al., 2005; Farnè & Ládavas, 2000).
